# Stakeholder controls and conflicts in research funding and publication

**DOI:** 10.1371/journal.pone.0264865

**Published:** 2022-03-09

**Authors:** Ralf C. Buckley

**Affiliations:** School of Environment & Sciences, Griffith University, Gold Coast, Australia; University of Toronto, CANADA

## Abstract

Academics are required by their university employers both to raise research funding and to publish research findings, but conditions imposed by research funders may conflict with the requirements of research publishers. These conflicts create risks, with potentially severe consequences, that differ between research fields and funders, and must be navigated by individual academics. I propose that universities report cases of conflict, including causes and resolutions, to national registries accessible to all research organisations. These could serve both as a warning to grant applicants, and a deterrent to future interference by funders.

## Introduction

Carrying out research can be costly, depending on topic and discipline. This gives research funders potential power over information, creating conundrums for researchers. McCrabb et al. [1] surveyed principal authors of articles cited in a medical review. Of the 104 who responded, 18% reported that research funders had attempted to influence publication of research results. McCrabb et al. concluded that research organisations should not accept funding under those conditions.

Here I argue that academics are routinely under pressure to seek out research funding from multiple sources, including those with political or commercial interests. Academics are also under pressure to publish their research findings, in journals whose publishers have legal and commercial interests. Universities operate within complex political and institutional contexts, including those associated with research funding and publication. To address the issues raised by McCrabb et al. [1] is not straightforward.

I focus here on the competing demands of research funders and publishers, as they affect individual academics. These stakeholder demands are themselves driven by larger-scale political arguments over, eg: the place of science in society, public access to published results of publicly-funded research [2, 3], and the social purposes of universities [4], but those are beyond the scope of this comment. I do not consider the interests of editors [5], only those of publishers. I do not address the dynamics of university ranking systems [6, 7], and their effects on external and internal funding structures [8, 9], such as differential strategic resource allocation to particular research fields or groups [10, 11]. My framework is practical rather than ethical or ideological: most university academics, as part of their job descriptions and performance criteria, are under pressure both to obtain research funding, and to publish research findings.

National competitive grant programs with minimal restrictions on publication, however, are far too small to fund everyone, and skewed towards a subset of individuals. Therefore, most academics are forced to apply for grants which do incorporate restrictions. These include: government grant programs that require industry partners; grants directly from individual government agencies and portfolios; and grants directly from private corporations, NGO’s, trusts and foundations. Here I consider the perspectives of the various stakeholders involved, and propose a possible policy response.

## Methods

This contribution is a Comment, based on qualitative information from the author’s own professional experience over half a century. That includes past positions in universities, government research agencies, and a large consulting company. University roles include: individual professor; dean of science; leader and director of large-scale cross-faculty and multi-university grant bids and research institutes; and director of research integrity. Other academic roles include: journal editor, reviewer and author; grant assessor nationally and internationally; member or chair of national and international advisory councils; and witness to judicial and parliamentary inquiries. Like many academics, I have also been threatened with litigation, and worse, over published findings and their reporting in mass media. Each of the cases, examples and issues referred to in this Comment is from the author’s involvement. Organisations and individuals are not named here, but are identifiable if necessary.

My focus here is on university academics. The same issues may also be faced by researchers in other organisations, but the job descriptions and management structures are different. For academics, position descriptions and promotion criteria explicitly require them to apply for external research funding, conduct and publish research, and contribute to public debate in their fields of expertise. Some academics also obtain funding via consultancies, but those are rarely publishable, because their topics are too narrow, their methods insufficiently rigorous, their data confidential to the client, and their theoretical contributions minimal. My focus here is on research funds labelled as grants and intended to generate publications.

## Research grant funders

There are many different types of research grants, each with their own risk and restrictions. Core funding for basic research in most developed nations is provided through national competitive grant programs, with overall budgets allocated by governments, and projects selected by semi-autonomous grant agencies, typically with annual application rounds and external expert assessors. These may cover all academic disciplines jointly, or they may be distinguished by major discipline, such as science, arts, humanities, or health. Examples include the US National Science Foundation, NSF, and the Australian Research Council, ARC. Data, intellectual property, and publication rights are generally assigned to grantees.

In many developed nations, governments also operate competitive grant programs that require end-user co-sponsors. These differ greatly in scale. Some are similar to pure-research grants, but with an applied focus and a partner contribution. Others are large multi-year consortia. These may be treated by industry partners as publicly-funded captured consultancies, used to provide competitive advantages to individual companies rather than to advance knowledge and national interests. From a researcher perspective, they all involve potential conflicts with co-funders and other sponsors and partners.

Some government agencies fund specialised research grant programs within their own portfolios. These are competitive, but often with political overtones, and they routinely contain restrictions on publication. Some private organizations also operate competitive research grant programs. Health insurers, for example, offer grants for research that may reduce their future costs. There are many private non-profit foundations that operate research grant programs, some very large. All of these may impose restrictions on publication.

Agencies responsible for allocating pure-research government grants do not need to consider co-funding, but only the efficient allocation of an overall budget between competing grant applications, so as to maximise reputable research outcomes. Their safest strategy, and the one they adopt in practice, is to fund applicants who already have successful track records. For grants that include end-user co-funding, the quantum of co-funding is also a primary consideration, since governments include those amounts in their political reporting of research funding. To reduce risks of political controversy, they prefer prior partners.

For end-user co-funders, in contrast, and also for grants funded by private organisations, NGOs, foundations, trusts, and government portfolio organisations, the risks and incentives are very different. Each of these has a strong commercial or political interest in particular research topics, and also in the specific outcomes of relevant research. They risk investing money in projects that may produce zero or negative return for themselves; and they have incentives to influence the reporting and publication of outcomes to produce or maximise positive returns.

## Equity issues

Pure-research grants from independent grant agencies have fewest restrictions, but total funds from these sources are limited, and distributed inequitably. For the most recently reported ARC Discovery (pure-research) round, funds allocated totalled ~15% of funds applied for. The number of projects funded was ~20% of the number of applications, and the funding per approved project was ~70% of funds requested. In addition, one of the principal evaluation criteria is success in previous applications. This creates positive feedback under which a small proportion of privileged academics run their research with few restrictions, but the remainder are forced to contend with potential conflicts.

The risks of having to navigate between the conflicting requirements of research funders and publishers, are thus distributed inequitably between academics. Those who received strong support at the commencement of their careers, and have established track records, may never face these dilemmas. They can rely on continual funding from national competitive grant agencies, with minimal restrictions on publication. Most researchers, however, have to seek funding from a wider range of sources, either because of historical career circumstances outside their control, or a focus on more applied topics or less well-funded disciplines. This provides an additional public-interest argument to reduce research funder influence or interference in publication.

This shortage of unrestricted research funding applies even more powerfully for academics in less wealthy developing nations, with limited centralised competitive grant funding. Even in developed nations, some governments use research funding as a political tool, by: reducing funding to independent grant agencies; redirecting funds via industry associations, captive NGOs, or programs directed by government staff; cancelling research grants already allocated; and restricting access to data held by government agencies. Researchers who are unable to rely on core pure-research grants are differentially forced to contend with all these considerations.

## Publication outlets

There is a wide range of options for publication and dissemination of research results. These include: articles, chapters, or books, subscription or open access, published by independent academic or commercial publishers; preprints posted to servers; data posted to repositories; graduate theses submitted to and held by universities; public or electronically-disseminated seminars or conference presentations; reports or monographs formally published by either the research institution, the primary research funder, or a co-funder; press releases, social and mass media reports, and articles in the popular press; and unpublished reports, either confidential or not. Each of these may or may not contain primary data, at various potential levels of detail, such as identification of individuals or geographical locations.

Most research funders require publication as a condition of a grant, and some also specify the type of outlet. For example, they may require researchers to provide the funder with a report including all data collected, or they may require an article in an open access journal, or an SCI/SSCI journal. Publication has legal meanings, eg in regard to intellectual property, data re-use, defamation, and liability. A commercial-in-confidence report is not publication, but if that report is then leaked or released, it may be considered to be in the public domain.

Each publication option has its own requirements. Publishing agreements with academic and professional societies, and commercial publishers, include warranties and indemnities from the authors. Online submission systems for journal publication include legal declarations related to funding, authorship, data, ethics, and use of previously published materials [12]. Higher-tier journals have more complex and stringent requirements, and more comprehensive checks and feedbacks. They send automated emails to co-authors, and they cross-reference global databases of funding agencies, ethics protocols, and publications. They use anti-plagiarism software, and check authors’ ORCID records. They do this to protect themselves against potential lawsuits and loss of reputation, both expensive.

## Funder-Publisher conflicts

Publication conflicts with research funding stakeholders occur principally for research grants where both grantees and grantors believe that they have rights to control research practice, data and publications, or where the demands of research funders conflict either with the requirements of journal publishers, or ethics protocols. These are most common for joint government-industry grant programs, and for grant programs run directly by government agencies, foundations, or private corporations. This is a key finding of McCrabb et al. [1].

Ways in which funders may influence publication include: refusal to permit publication at all; attempts to influence publication narrative or wording; addition, removal or substitution of authors; parallel publication without acknowledgement; the requirement to publish results as a funder report; a demand to publish in a particular journal category, or a particular academic discipline; a demand to control issue of any press releases or project publicity; refusal to be acknowledged as a research funder; and refusal for data to be posted to a repository.

Some funding agreements include a clause that the researcher must seek the funder’s permission before publishing results; and sometimes this permission is withheld, either completely, or until wording, authorship, or publication outlet is changed. Some funders attempt to impose demands that conflict with publisher requirements, eg as regards authorship, acknowledgement of funding, or access to data and analyses. In such cases, if no agreement can be reached, researchers may be excluded from publishing in high-tier journals. These demands commonly arise when a research project has produced results that are commercially or politically unpalatable to the funder.

These politics, however, are not always immediately transparent. The economic value of parks via improved mental health of visitors using public infrastructure, for example, far exceeds that via commercial tourism [13]. This research was politically inconvenient for one of the co-funders, which wants to grant exclusive tourism development rights inside parks, and they have attempted to avoid association with future publications. That was unexpected. The other co-funder, a parallel organisation in another jurisdiction, had no objections.

Some research funders demand control over raw data. Such demands can take many different forms. They may argue that they own those data, and refuse to allow them to be made available as part of journal publication, eg as supplementary materials or via public data repositories. Alternatively, they may demand access to personal or other confidential data collected during a research project, such as individual health conditions, socioeconomic status, or political opinions. This generally conflicts with research ethics protocols requiring confidentiality. A funding agency with commercial interests may potentially make unethical use of confidential personal data, or allow unauthorized release to third parties.

Separately from raw data, funders may demand rights to intellectual property, particularly commercializable IP. Universities are increasing pressure to commercialise their research [14]. They generally own IP produced by academic staff, though not students. Some research funders demand that graduate students supported by scholarships or project funding, must sign over IP before starting. Conflicts also arise if a funder refuses to allow use of project IP in future research, effectively stalling a line of investigation. In countries where government funding to universities is calculated partly on patents, there is pressure to register as many patents as possible, even if they are worthless or unenforceable. There is thus a risk that the funding agreement for one project may block progress on future research.

## Risks and dilemmas for researchers

Universities routinely provide assistance to individual academics in negotiating funding agreements, through offices for research grants, commercial enterprise, and legal services. All of these are very valuable. At the same time, however, universities evaluate academic performance through research funding raised, as well as research publications produced. Both for promotion and performance review, many academics are required, by formal position descriptions, to win substantial external funding. Grants are thus valuable to researchers, even if conflicts with funders force results to remain unpublished. From an education policy perspective, however, that represents a waste of taxpayer resources, not only in government contributions to grants, but also in salaries and support. Therefore, mechanisms to avoid or resolve conflicts with funding stakeholders over research publication are important.

These conflicts generally do not arise from failure to read the fine print in funding agreements, nor from uncertainties at the time applications are lodged. Funders commonly require applicants to accept the terms of a funding agreement at the time of lodgement, and research organisations are familiar with conditions, and careful to consider potential problems. Rather, these possible conflicts are one of a number of risks that researchers must navigate, in order to be able to carry out research at all. Universities can ask funders for modifications to agreements, either at application or at acceptance, but most funders refuse even the smallest changes. Their attitude is, take it or leave it, we have plenty of other applications. Even if a researcher’s university reiterates the potential risks of publication interference, this is overridden by the pressure on individuals to apply for research funds.

Once research is complete and ready for publication, researchers must identify and navigate any potential conflicts between funders and publishers. Publishers are powerful. If authors do not comply with publisher requirements, their manuscripts are simply not considered. For higher-tier journals, authors are prohibited from releasing results until a publication embargo date is reached. Authors must also declare any potential conflicts of interest, and certify that their research was carried out rigorously, without reference to external influences. In practice this means that for high-tier journals, authors cannot send draft manuscripts to funders.

If funding agreements give funders a right to vet or control publications, then researchers face a dilemma. If their manuscripts are aimed at lower-tier journals, they can choose whether or not to send drafts to funders. For high-tier journals, however, this option is not available. They can only go ahead and publish, and face any risk from funders subsequently. They must estimate probabilities firstly, that any of the funders may be dissatisfied, and secondly, that they might take any action. Funders have a substantial arsenal, including injunctions, breach of contract litigation, and blacklisting of researchers or institutions from any future grants.

If funders do take any action, researchers must consider their potential defences. Most publishing agreements contain indemnities, so researchers could potentially find themselves defending any actions by funders against publishers. Universities may defend their staff, but not always. Codes of conduct for staff may require academics to obtain formal approval before submitting any article for external publication, providing universities with a legal escape route from liability; but there is rarely any practical mechanism for this. Potentially, therefore, these dilemmas may have substantial consequences for individual academics, even if they are rare.

In the hierarchy of risks faced by university academics, this one may be underrated. More visible risks include investing time and effort in grant applications and manuscripts that are rejected, or in research projects that do not yield publishable results. Less frequent risks include unauthorised publication of one’s data by colleagues or reviewers, and plagiarism of publications. McCrabb et al. [1], however, showed that conflicts with funders are in fact quite frequent, an invisible undercurrent to routine research. They did not report what steps researchers had taken to resolve these conflicts, so we cannot judge to what degree published results may reflect funder demands. That would seem to be a priority for future research.

## Discussion: National registries as a possible response

The results reported by McCrabb et al. [1] indicate that government agencies other than national research grant agencies, and mixed-source grants, generated the highest numbers of reported cases, and that industry funding did not generate any. They do not report, however, how many grants from each funder type, respondents had actually received. For example, perhaps none of the respondents had received funding from industry sole sources. Comparable differences between employers, rather than funders, are reported by Driscoll et al. [15].

McCrabb et al. [1] proposed 8 potential measures to address the issue of funder interference with publication. Some already exist: research ethics committees, academic employee codes of conduct, journal codes of ethics [12], and declarations of funding and conflict in individual articles. McCrabb et al. proposed that all of these should explicitly address, and publish, the terms of funding agreements. Most of these terms, however, are already available on funder websites. McCrabb et al. also proposed that government agencies should remove clauses from research funding agreements that require approval of results prior to publication, and that universities should refuse funding that includes such restrictions; but these are unlikely.

McCrabb et al. [1] also suggested that universities should set up mechanisms to report and publicise suppression. This is feasible and, I suggest, well worthwhile. Perhaps the most powerful approach would be to establish national registries of documented cases, categorized by type and listed by funder, and accessible to all research organisations. This would serve both as a warning to funding applicants, and a deterrent to funders wanting to influence future publications. Researchers could check routinely whether particular funders have a disproportionate record of unwarranted interference, and could analyse patterns across funders, at regular intervals. A registry would go beyond the wording of grant agreements, to show what steps funders have actually taken when conflicts arose.

To be effective, such a registry would need safeguards to ensure the accuracy of information posted. For example, governments could require universities to report publication conflicts with research funders, as an addition to their other routine reporting requirements. The formal process could be initiated by the individual researchers affected, checked by research grants and legal services offices, and posted to the repository, with supporting information, by a central university officer with adequate legal support. To avoid political abuse, the repository could be available only to research organisations, not the general public.

Such a repository would have little effect initially, but if it were analysed annually, it would show which funders regularly exercise vested economic or political interests, and which groups of academics are most affected. That is, it would provide data for future analyses complementary to that of McCrabb et al. [1], but with a greater degree of detailed documentation. These in turn would exert a semi-public counter-pressure on funders, to permit proper publication free from interference, in a similar way to consumer reports, and trade-practices or health-and-safety litigation. We do not want to discourage organisations from contributing to research funding; but we do want them to recognise that research is only valuable if conducted and published independently, and that research funding is not a cheap backdoor consultancy, but a contribution to knowledge.
